# Supplementary material for: Changes in the estimated glomerular filtration rate and predictors of the renal prognosis in Japanese patients with type 2 diabetes: A retrospective study during the 12 months after the initiation of tofogliflozin
Source: PLoS One. 2023 Sep 21;18(9):e0292014. doi: 10.1371/journal.pone.0292014 (PMC10513294; doi:10.1371/journal.pone.0292014)
Supplement: Attachment — Submitted filename: Plos One eGFR and tofogliflozin Manuscript Review.docx [file pone.0292014.s003.docx]

Manuscript Review	29 March 2013

PLOS ONE

**Changes in the estimated glomerular filtration rate and predictors of the renal prognosis in Japanese patients with type 2 diabetes: A retrospective study during the 12 months after the initiation of tofogliflozin.**

The study investigated the renal protective effects of tofogliflozin over a 1-year period. The manuscript is well-written and has a good flow. This study adds vital data on the drug in human subjects as only animal studies have investigated the effect on eGFR to date. For a drug with a short half-life, it is important to demonstrate that it can achieve the goal of preserving renal function in patients with diabetes.

Comments to authors:

1. Line 38 – 39: authors mention a negative eGFR correlation with baseline eGFR and HbA1c. Does this mean if a patient had a baseline eGFR of 100, they were likely to have an eGFR below 100 at 12 months (negative correlation)? How does this translate to a ‘protective’ effect?
2. Authors to mention and clarify the male preponderance (71%); is this reflective of DM II demographic in Japan or due to clinical criteria for tofogliflozin treatment?
3. Table 2: month 3,6 and 9 do not show any significant trends and can be omitted from the table to allow space to put actual p-values instead of ** or #
4. Line 190 – 191: the authors state that weight and systolic blood pressure gradually decreased over 12 months in both groups. The over-all weight loss in both groups is 3kg over 12 months and SBP is 4 and 7 mmHg, which are both modest changes. Authors should probably state this actual weight loss and SBP change averages per group instead of a general description. The main changes occurred in the first month and then plateaued to the end i.e. not really a trend over time.
5. Line 198 – 199: authors mention “in uPE and urinary L-FABP were larger in the low-eGFR group than in the normal GFR group, there were no significant differences” Please clarify statistical significance versus clinical significance when discussing result findings, throughout the manuscript. Readers should get clarity on the potential clinical benefit of the drug based on whether the stipulated results show a clinically meaningful change; p-values can be <0.001 but with negligible actual mean differences. This must be clearly discussed for the important parameters including end-point eGFR, weight, SBP, uric acid findings. Second example: line 221 – 223 “*change in the eGFR (0.2±6.0 mL/min/1.73 m2) was significantly 222 (P=0.02) improved after tofogliflozin administration compared to before administration 223 (-3.6±5.5 mL/min/1.73 m2)”.*
6. Statistics: Which least squares method (Linear, Weighted, Robust or Nonlinear) was used, and why. Were there any outliers: if yes, how were they handled?
7. The short title does not indicate the central factor, which is the drug, tofogliflozin.

Dr T Jalavu

30 March 2023
