# Supplementary material for: Changes in the estimated glomerular filtration rate and predictors of the renal prognosis in Japanese patients with type 2 diabetes: A retrospective study during the 12 months after the initiation of tofogliflozin
Source: PLoS One. 2023 Sep 21;18(9):e0292014. doi: 10.1371/journal.pone.0292014 (PMC10513294; doi:10.1371/journal.pone.0292014)
Supplement: Attachment — Submitted filename: Responses to reviewers.docx [file pone.0292014.s004.docx]

**Responses to the academic editor**

1. The lack of clarity regarding whether it was investigator initiated or pharma driven

**Response:** We added the text in the **Method** section.

2. The absence of an apriori statistical analysis plan to predict sample sizes needed

**Response:** The sample sizes required to compare the changes in eGFR between the normal- and low-eGFR groups were 259 in the present study. The statistical power was calculated to be 0.51 according to the subject’s number of the FAS. Therefore, the sample size was too small to compare the changes in eGFR between the two groups. We added the text showing the present study's low statistical power in the revised manuscript's limitations.

3. The retrospective analysis and small sample size

**Response:** Although these limitations were mentioned in the initial submission version, we amended the text with more emphasis in the revised manuscript.

4. The short follow up time

5. The relevance of the comparison of a smaller group with 12 months follow up prior to commencing the drug

**Response:** We added the text as another limitation of this study in the revised manuscript. Although a more extended observation was desirable, as the editor pointed out, it isn’t easy to accurately evaluate the effects of tofogliflozin because drugs other than tofogliflozin are often changed in actual clinical practice.

6. The erroneous conclusions drawn by the investigators - as per my comments

**Response:** We weakened the tone of the conclusion of the revised manuscript.

**Responses to the reviewers**

1. Line 38 – 39: authors mention a negative eGFR correlation with baseline eGFR and HbA1c. Does this mean if a patient had a baseline eGFR of 100, they were likely to have an eGFR below 100 at 12 months (negative correlation)? How does this translate to a ‘protective’ effect?

**Response:** We amended the **Conclusions** of the **Abstract** after adding the actual value of the initial dip to the **Results**.

2. Authors to mention and clarify the male preponderance (71%); is this reflective of DM II demographic in Japan or due to clinical criteria for tofogliflozin treatment?

**Response:** The actual male/female ratio of Japanese patients with type 2 diabetes is unknown because Japan has no registration system. Our previous cross-sectional reports (Nephrol Dial Transplant 25: 1161, 2010, J Diabates Investig 1: 273, 2010, BMC Nephrol 13: 48, 2012, Nephron Extra 3: 66, 2013, J Diabetes Complications 26: 286, 2012, Diabetes Care 37: e7, 2014, Geriatr Gerontol Int 17: 24, 2017, etc.) demonstrated that 60-62% of outpatients with type 2 diabetes were men, and the proportion in the present study (71%) appears to be higher in comparison to those reports. The proportions of men were also 71-73% in our reports that investigated the effects of SGLT2 inhibitors in patients with type 2 diabetes (Diabetes Metab Syndr Obes 12: 1783, 2019, PLoS One 16: e0248577, 2021), and it seems similar to that in the present study.

No criteria for the indication of tofogliflozin treatment were established in the present study because it was not randomized. A possible reason for this high ratio of male subjects may be the avoidance of patients who may develop genital urinary tract infections, an adverse event of SGLT2 inhibitors. In this study, 17 of 22 (77%) patients with cerebrovascular disease and 22 of 24 (92%) patients with coronary heart disease were men. In such cases, SGLT2 inhibitors, which have been reported to be effective for secondary prevention, might have been positively selected. It is considered that these circumstances combined to cause the difference in the male/female ratio between the present study and our previous investigations.

We included considerations regarding the male/female ratio in the limitation of the **Discussion** section and text showing that the subjects of this study did not reflect the general clinical characteristics of Japanese patients with type 2 diabetes.

3. Table 2: month 3,6 and 9 do not show any significant trends and can be omitted from the table to allow space to put actual p-values instead of ** or #

**Response:** We reconstructed Table 2 as **Appendix 1**. As the reviewer pointed out in the following paragraph, the changes in the clinical parameters did not necessarily show the same course. For example, the blood pressure did not change significantly after 3 months, although the body weight and HbA1c gradually decreased up to 6 months. It doesn’t seem easy to understand these trends from **Appendix 1**. Therefore, we would like to keep this table without deleting the values at 3, 6, and 9 months. The P values for comparing the changes between the normal-eGFR and low-eGFR groups are shown in the text of the revised manuscript. In addition, the errors in the baseline body weight value were fixed.

4. Line 190 – 191: the authors state that weight and systolic blood pressure gradually decreased over 12 months in both groups. The over-all weight loss in both groups is 3kg over 12 months and SBP is 4 and 7 mmHg, which are both modest changes. Authors should probably state this actual weight loss and SBP change averages per group instead of a general description. The main changes occurred in the first month and then plateaued to the end i.e. not really a trend over time.

**Response:** We amended the text that the reviewer pointed out.

5. Line 198 – 199: authors mention “in uPE and urinary L-FABP were larger in the low-eGFR group than in the normal GFR group, there were no significant differences” Please clarify statistical significance versus clinical significance when discussing result findings, throughout the manuscript. Readers should get clarity on the potential clinical benefit of the drug based on whether the stipulated results show a clinically meaningful change; p-values can be <0.001 but with negligible actual mean differences. This must be clearly discussed for the important parameters including end-point eGFR, weight, SBP, uric acid findings. Second example: line 221 – 223 “change in the eGFR (0.2±6.0 mL/min/1.73 m2) was significantly 222 (P=0.02) improved after tofogliflozin administration compared to before administration 223 (-3.6±5.5 mL/min/1.73 m2)”.

**Response:** We amended the first and second paragraphs in the **Discussion** section in accordance with the reviewer’s suggestion.

6. Statistics: Which least squares method (Linear, Weighted, Robust or Nonlinear) was used, and why. Were there any outliers: if yes, how were they handled?

**Response:** The present study used the linear least square method without the exclusion of outliers after confirming that these values were not caused by transcription errors when they were transcribed from the medical records. Even when the outliers assessed by boxplots were excluded, the statistical results did not change, as shown in **Appendix 2** (differences from Table 4 are shown in red).

We added the statistical method and results to the **Methods** and **Results** sections.

In addition, there was an error in the correlation coefficient between the change in eGFR and the uric acid level in the initial submission version, so 1.117 was changed to 0.017.

7. The short title does not indicate the central factor, which is the drug, tofogliflozin.

**Response:** We amended the short title.

Appendix 1: Table 2 after excluding the values at 3, 6, and 9 months (P values added)

|  | Baseline | 1 month | P† | 12 months | P† | Change from baseline | P‡ |
| --- | --- | --- | --- | --- | --- | --- | --- |
| Body weight (kg) |  |  |  |  |  |  |  |
| All subjects (*n*=91) | 74.3±14.7 | 72.9±14.5 | <0.01 | 71.5±14.7 | <0.01 | -2.9±3.5 |  |
| Normal-eGFR (*n*=63) | 73.8±15.3 | 72.5±15.3 | <0.01 | 71.1±15.3 | <0.01 | -2.8±3.8 |  |
| Low-eGFR (*n*=28) | 75.4±13.3 | 73.9±12.8 | <0.01 | 72.4±13.5 | <0.01 | -3.0±2.7 | 0.78 |
| Systolic blood pressure (mmHg) |  |  |  |  |  |  |  |
| All subjects (*n*=122) | 135±15 | 131±13 | <0.01 | 131±14 | <0.01 | -5±14 |  |
| Normal-eGFR (*n*=82) | 134±12 | 130±12 | 0.02 | 130±13 | 0.01 | -4±13 |  |
| Low-eGFR (*n*=40) | 139±18 | 133±15 | 0.03 | 132±16 | 0.01 | -7±16 | 0.36 |
| Diastolic blood pressure (mmHg) |  |  |  |  |  |  |  |
| All subjects (*n*=119) | 80±13 | 78±11 | 0.09 | 79±12 | 0.40 | -2±12 |  |
| Normal-eGFR (*n*=80) | 80±12 | 78±11 | 0.06 | 80±12 | 0.99 | -0±11 |  |
| Low-eGFR (*n*=39) | 79±16 | 78±12 | 0.77 | 77±12 | 0.09 | -2±9 | 0.32 |
| uPE (mg/dL) |  |  |  |  |  |  |  |
| All subjects (*n*=121) | 35.5±76.9 | 18.6±47.6 | <0.01 | 11.8±37.1 | <0.01 | -23.6±58.7 |  |
| Normal-eGFR (*n*=81) | 20.5±49.2 | 9.3±26.3 | <0.01 | 6.4±24.2 | <0.01 | -14.1±35.3 |  |
| Low-eGFR (*n*=40) | 65.8±108.8 | 37.5±70.8 | <0.01 | 22.9±53.4 | <0.01 | -42.9±86.5 | 0.12 |
| uACR (mg/gCr) |  |  |  |  |  |  |  |
| All subjects (*n*=101) | 224.8±644.2 |  |  | 151.0±438.5 | <0.01 | -73.7±259.1 |  |
| Normal-eGFR (*n*=71) | 93.3±273.9 |  |  | 80.9±271.8 | 0.15 | -12.0±80.8 |  |
| Low-eGFR (*n*=30) | 535.8±1052.5 |  |  | 316.9±666.6 | <0.01 | -218.8±429.8 | <0.01 |
| Urinary L-FABP (μg/gCr) |  |  |  |  |  |  |  |
| All subjects (*n*=58) | 12.0±26.0 |  |  | 9.8±26.5 | 0.02 | -2.1±9.1 |  |
| Normal-eGFR (*n*=39) | 9.7±28.9 |  |  | 9.1±31.2 | 0.28 | -0.6±6.0 |  |
| Low-eGFR (*n*=19) | 16.6±18.9 |  |  | 11.3±12.6 | 0.03 | -5.2±13.2 | 0.14 |
| HbA1c (%) |  |  |  |  |  |  |  |
| All subjects (*n*=129) | 8.4±1.5 | 7.8±1.2 | <0.01 | 7.4±1.0 | <0.01 | -0.9±1.3 |  |
| Normal-eGFR (*n*=86) | 8.4±1.6 | 7.9±1.3 | <0.01 | 7.3±0.9 | <0.01 | -1.0±1.3 |  |
| Low-eGFR (*n*=43) | 8.3±1.4 | 7.8±1.0 | <0.01 | 7.6±1.1 | <0.01 | -0.7±1.1# | 0.06 |
| Hemoglobin (g/L) |  |  |  |  |  |  |  |
| All subjects (*n*=130) | 143±15 | 145±15 | <0.01 | 147±16 | <0.01 | 4±9 |  |
| Normal-eGFR (*n*=87) | 144±14 | 146±15 | <0.01 | 148±17 | <0.01 | 4±10 |  |
| Low-eGFR (*n*=43) | 142±15 | 143±15 | 0.50 | 145±15 | 0.03 | 2±9 | 0.36 |
| Uric acid (μmol/L) |  |  |  |  |  |  |  |
| All subjects (*n*=118) | 305±70 | 295±70 | 0.02 | 286±63 | <0.01 | -18±50 |  |
| Normal-eGFR (*n*=78) | 297±71 | 289±75 | 0.12 | 276±65 | <0.01 | -21±49 |  |
| Low-eGFR (*n*=40) | 319±67 | 306±59 | 0.10 | 305±53 | 0.09 | -13±54 | 0.65 |
| eGFR (mL/min/1.73 m^2^) |  |  |  |  |  |  |  |
| All subjects (*n*=130) | 68.7±21.8 | 65.3±21.9 | <0.01 | 67.5±21.1 | 0.19 | -1.2±8.2 |  |
| Normal-eGFR (*n*=87) | 80.6±15.0 | 76.3±16.9 | <0.01 | 78.7±14.4 | 0.15 | -1.9±9.0 |  |
| Low-eGFR (*n*=43) | 44.6±10.4 | 43.1±11.3 | <0.01 | 44.8±12.3 | 0.76 | 0.2±6.0 | 0.44 |

eGFR, estimated glomerular filtration rate; uPE, urinary protein excretion; uACR, urinary albumin-to-creatinine ratio; L-FABP, liver-type fatty acid-binding protein

† P: vs. corresponding value at baseline, ‡ P: vs. corresponding value in the normal-eGFR group.

Appendix 2; Table 4 after excluding outliers

|  | All subjects (*n*=130) | | | | Normal eGFR (*n*=87) | | | | Low eGFR (*n*=43) | |
| --- | --- | --- | --- | --- | --- | --- | --- | --- | --- | --- |
|  | Single regression | | Multiple regression | | Single regression | | Multiple regression | | Single regression | |
|  | β | *P* | β | *P* | β | *P* | β | *P* | β | *P* |
| Male sex | 2.523 | 0.11 |  |  | 4.246 | 0.06 |  |  | -2.273 | 0.29 |
| Age (/years) | 0.003 | 0.96 |  |  | -0.009 | 0.91 |  |  | -0.085 | 0.37 |
| Duration of diabetes (/years) | -0.098 | 0.31 |  |  | -0.141 | 0.28 |  |  | -0.065 | 0.61 |
| Smoking history | 0.949 | 0.52 |  |  | 2.243 | 0.26 |  |  | -2.268 | 0.24 |
| Current drinker | -0.113 | 0.94 |  |  | -1.006 | 0.64 |  |  | 3.109 | 0.17 |
| Body weight (/kg) | 0.068 | 0.22 |  |  | 0.115 | 0.13 |  |  | -0.035 | 0.64 |
| Body mass index (/kg/m^2^) | 0.087 | 0.59 |  |  | 0.010 | 0.62 |  |  | 0.005 | 0.98 |
| Systolic blood pressure (/mmHg) | -0.057 | 0.30 |  |  | -0.100 | 0.22 |  |  | -0.024 | 0.70 |
| Diastolic blood pressure (/mmHg) | -0.032 | 0.61 |  |  | -0.054 | 0.53 |  |  | 0.029 | 0.71 |
| Diabetic retinopathy | 1.239 | 0.49 |  |  | 0.826 | 0.75 |  |  | 1.132 | 0.60 |
| Diabetic peripheral neuropathy | 1.447 | 0.40 |  |  | 1.802 | 0.45 |  |  | 0.351 | 0.88 |
| Cerebrovascular disease | -0.667 | 0.73 |  |  | 0.295 | 0.91 |  |  | -2.680 | 0.24 |
| Coronary heart disease | 2.890 | 0.12 |  |  | 4.927 | 0.06 |  |  | -0.735 | 0.74 |
| Obesity | 0.419 | 0.79 |  |  | 1.443 | 0.50 |  |  | -2.516 | 0.24 |
| Hypertension | -0.233 | 0.90 |  |  | -1.064 | 0.79 |  |  | 0.875 | 0.79 |
| Albuminuria | -1.187 | 0.44 |  |  | -2.092 | 0.33 |  |  | -1.791 | 0.39 |
| Hyperuricemia | -0.660 | 0.67 |  |  | 0.447 | 0.85 |  |  | -3.049 | 0.10 |
| RAAS inhibitors use† | -0.263 | 0.86 |  |  | 0.263 | 0.89 |  |  | -2.639 | 0.17 |
| Calcium channel blockers use | 1.457 | 0.32 |  |  | 0.641 | 0.75 |  |  | 1.763 | 0.35 |
| Urate-lowering agents use | -1.167 | 0.38 |  |  | -0.933 | 0.73 |  |  | -4.032 | 0.03 |
| Metformin use | 2.976 | 0.04 | 3.188 | 0.02 | 4.417 | 0.03 | 2.317 | 0.22 | 1.470 | 0.43 |
| Sulfonylureas use | 5.185 | 0.08 |  |  | 7.451 | 0.07 |  |  | 1.100 | 0.76 |
| Thiazolidinediones use | -2.376 | 0.49 |  |  | -1.676 | 0.69 |  |  | -3.718 | 0.55 |
| α-glucosidase inhibitors use | 0.985 | 0.77 |  |  | 1.756 | 0.65 |  |  |  |  |
| Glinides use | -1.726 | 0.72 |  |  | -1.027 | 0.85 |  |  |  |  |
| DPP-4 inhibitors use | 0.581 | 0.69 |  |  | 0.126 | 0.95 |  |  | 0.731 | 0.70 |
| GLP-1 receptor agonists use | 2.001 | 0.37 |  |  | 3.248 | 0.29 |  |  | -0.497 | 0.86 |
| Insulin use | -0.623 | 0.73 |  |  | -1.451 | 0.60 |  |  | -0.679 | 0.74 |
| uPE (/mg/dL) | 0.054 | 0.21 |  |  | 0.089 | 0.20 |  |  | -0.084 | 0.23 |
| uACR (/mg/gCr) | -0.002 | 0.94 |  |  | -0.015 | 0.69 |  |  | 0.034 | 0.54 |
| Urinary L-FABP (/μg/gCr) | 0.167 | 0.64 |  |  | 0.145 | 0.77 |  |  | -0.079 | 0.88 |
| HbA1c (/%) | -1.430 | <0.01 | -1.148 | 0.01 | -2.179 | <0.01 | -1.640 | <0.01 | 0.462 | 0.49 |
| Hemoglobin (/g/L) | 0.298 | 0.55 |  |  | 0.643 | 0.35 |  |  | -0.251 | 0.68 |
| Uric acid (/μmol/L) | 0.017 | 0.09 |  |  | 0.012 | 0.39 |  |  | 0.027 | 0.05 |
| eGFR (/mL/min/1.73 m^2^) | -0.102 | <0.01 | -0.101 | <0.01 | -0.219 | <0.01 | -0.174 | <0.01 | 0.035 | 0.70 |
